# Effect of GLP-1RA Treatment on Adhesion Molecules and Monocyte Chemoattractant Protein-1 in Diabetic Patients with Atherosclerosis

**DOI:** 10.3390/life14060690

**Published:** 2024-05-28

**Authors:** Marcin Hachuła, Marcin Basiak, Michał Kosowski, Bogusław Okopień

**Affiliations:** Department of Internal Medicine and Clinical Pharmacology, Medical University of Silesia, Medyków 18, 40-752 Katowice, Poland; marcin.hachula@gmail.com (M.H.); mkosowski@sum.edu.pl (M.K.); bokopien@sum.edu.pl (B.O.)

**Keywords:** atherosclerosis, GLP-1 receptor agonist, diabetes mellitus, MCP-1, ICAM-1, semaglutide, dulaglutide

## Abstract

Cardiovascular disease (CVD) remains a prominent cause of global mortality, primarily driven by atherosclerosis. Diabetes mellitus, as a modifiable risk factor, significantly contributes to atherogenesis. Monocyte recruitment to the intima is a critical step in atherosclerotic plaque formation, involving chemokines and adhesion molecules such as selectins, ICAM-1, and MCP-1. Glucagon-like peptide 1 receptor agonists (GLP-1RAs) are a promising group of drugs for reducing cardiovascular risk in diabetic patients, prompting investigation into their mechanisms of action. This interventional study enrolled 50 diabetes patients with atherosclerotic plaque, administering GLP-1RA for 180 days. Serum concentrations of MCP-1, ICAM-1, and L-selectin were measured before and after treatment. Anthropometric and biochemical parameters were also assessed. GLP-1RA treatment resulted in significant improvements in anthropometric parameters, glycemic control, blood pressure, and biochemical markers of liver steatosis. Biomarker laboratory analysis revealed higher baseline levels of MCP-1, ICAM-1, and L-selectin in diabetic patients with atherosclerotic plaque compared to healthy controls. Following treatment, MCP-1 and L-selectin levels decreased significantly (*p* < 0.001), while ICAM-1 levels increased (*p* < 0.001). GLP-1RA treatment in diabetic patients with atherosclerotic plaque leads to favorable changes in serum molecule levels associated with monocyte recruitment to the endothelium. The observed reduction in MCP-1 and L-selectin suggests a potential mechanism underlying GLP-1RA-mediated cardiovascular risk reduction. Further research is warranted to elucidate the precise mechanisms and clinical implications of these findings in diabetic patients with atherosclerosis.

## 1. Introduction

Cardiovascular disease (CVD) remains the foremost global cause of death, as indicated by the Global Burden of Disease (GBD) report, which estimated 19.8 million fatalities attributed to CVD worldwide in 2022 [1]. The main mechanism in the pathogenesis of cardiovascular disease is atherosclerosis [2]. The development of atherosclerosis is multifactorial and consists of numerous non-modifiable and modifiable risk factors. These factors are collectively referred to as cardiovascular risk factors. One of the modifiable risk factors is diabetes mellitus [3]. Excessive blood sugar levels and glycemic fluctuations occurring in diabetes, which result in an increase in the concentration of reactive oxygen species, are strongly associated with atherogenesis, playing a pivotal role not only in triggering the development of atherosclerotic plaques but also exacerbating their advancement and vulnerability [4]. Cardiovascular diseases are the most prevalent cause of mortality and morbidity among individuals with diabetes [5]. A particularly crucial step in the formation of atherosclerotic plaque is the process of monocyte recruitment to the intima. This process includes the rolling, adhesion, activation, and transmigration of monocytes. Numerous chemokines and adhesion molecules participate in these phases [6].

Rolling is facilitated by the selectin family; they are directly involved in atherosclerotic plaque formation [7]. The P and E selectins are expressed by inflamed endothelial cells; their role in the development of atherosclerosis is very well understood [8,9]. In contrast, L-selectin is expressed in the majority of leukocytes and its involvement in this process is still being studied [10].

The binding of selectins with their ligands enables leukocytes to adhere to inflamed sites in the endothelium. In atherosclerotic plaque, chronic inflammation leads to the expression of adhesion molecules and their promotion at the surface of the endothelium. One of these adhesion molecules is intercellular adhesion molecule-1 (ICAM-1), which is engaged in the adhesion of leukocytes and accelerates the migration of these cells into the intima [11]. The involvement of adhesive molecules such as ICAM-1 in cardiovascular diseases is still being studied; however, there is some evidence that their levels are higher in patients with a higher risk of cardiovascular diseases [12,13].

In monocyte transmigration, the predominant chemokine is monocyte chemotactic protein-1 (MCP-1). Its leading role is to recruit monocytes, favoring their transendothelial migration from the circulation to the intima, thus leading to the progression of atherosclerotic plaque [14]. The involvement of MCP-1 in the development of atherosclerosis has been widely recognized and supported by numerous preclinical models in animals, experimental studies on humans with the presence of atherosclerotic plaque in the carotid and coronary arteries, as well as in cases of its instability [15].

Scientists worldwide have recently begun dedicating their attention to a novel class of antidiabetic drugs known as glucagon-like peptide 1 receptor agonists (GLP-1RA). Several important randomized clinical trials, like SUSTAIN, LEADER, REWIND, and PIONEER, have shown that diabetes patients who undergo GLP-1RA therapy have a lower risk of cardiovascular events [16,17]. Recently, both the ESC Guidelines on cardiovascular disease prevention and the ADA have endorsed the use of GLP1-RAs in patients with diabetes and atherosclerotic cardiovascular disease (ASCVD) to mitigate cardiovascular-renal outcomes [18,19].

Regrettably, there is currently an absence of studies that firmly clarify the mechanism by which this group of drugs significantly reduces cardiovascular risk. A hypothesis has arisen claiming that these drugs have an influence on several steps in the process of atherogenesis, which leads to a reduction in cardiovascular events in individuals diagnosed with ASCVD.

The aim of our study was to investigate this hypothesis and verify whether GLP-1 receptor agonists influence the concentrations of chemokines and adhesion molecules involved in the progression of atherosclerotic plaque, such as MCP-1, L-selectin and ICAM-1 in a group of individuals with diabetes mellitus type 2 and confirmed atherosclerotic plaque in the carotid artery.

## 2. Materials and Methods

The medical experiment was conducted between January 2022 and September 2023. We enrolled 50 participants, aged between 41 and 81 (mean age of 60.7) from a total of 91 patients for our study. All participants were diagnosed with type 2 diabetes mellitus, dyslipidemia and had confirmed the presence of atherosclerotic plaque in the ultrasound Doppler study of carotid arteries. Only those who met the criteria for inclusion and passed very stringent and limited exclusion criteria were eligible for entry into the study. Informed consent was given by each patient in accordance with the Declaration of Helsinki. The information pertaining to the subjects was anonymized. Patients were enrolled at the Department of Internal Medicine and Clinical Pharmacology in Katowice, Poland, as well as through referrals from diabetic outpatient clinics. The study protocol received approval from the Bioethical Committee of the Medical University of Silesia (PCN/CBN/0052/KB1/45/I/22). In a study, 16 patients were given semaglutide and 34 patients were given dulaglutide, both at a standard dose for lowering blood glucose levels. For semaglutide, patients initiated treatment with a dose of 0.25 mg, which was increased to 0.5 mg after 4 weeks, and then to 1 mg after another 4 weeks. Exceptions were made for patients who reported an increase in adverse events at the 1 mg dose; in such cases, the dosage was reduced to 0.5 mg. Additionally, patients who achieved significant improvement in their glycemic profile at the 0.5 mg dose were not eligible for a dose increase. Conversely, for dulaglutide, the target dose was 1.5 mg, with an option to increase to 3 mg in the case of inadequate glycemic control while tolerating adverse events. The medication was administered on a weekly basis, consistently at the same time each day. Throughout the intervention, there were no modifications made to the GLP-1 receptor agonist therapy or any other drugs. The intervention lasted for a period of 26 weeks.

### 2.1. Inclusion and Exclusion Criteria

The requirements for inclusion in the study were as follows: a diagnosis of diabetes mellitus type 2, dyslipidemia, which was defined as having serum total cholesterol (TC) levels above 190 mg/dL, HDL cholesterol lower than 40 mg/dL and triglyceride (TG) levels above 150 mg/dL or were undergoing treatment for dyslipidemia, and the presence of atherosclerotic plaque in the carotid artery. Participants were ineligible for the study if they had the following: recognized type 1 diabetes, exacerbation of chronic heart failure or if they had undergone percutaneous coronary intervention (PCI), coronary artery bypass grafting (CABG), or experienced a stroke within 3 months prior to the study. Additionally, patients with unstable angina were also excluded. The exclusion criteria contained acute and chronic pancreatitis, acute exacerbations of autoimmune disorders, uncontrolled thyroid disease manifested by abnormal TSH values, and any other acute inflammatory processes (including COVID-19 infection) within 4 weeks prior to the study. The reason for exclusion from participation in the study also included acute infections characterized by an increase in CRP value of 5 mg/dL or significant leukocytosis. Additionally, patients with a glomerular filtration rate (eGFR) below 45 mL/min/1.73 m^2^, classified as chronic kidney disease (CKD) stage G3b, acute and chronic liver diseases (indicated by transaminase levels exceeding 3 times the norm), or a medical history of diagnosed chronic viral hepatitis were also excluded. Lack of informed consent, alcoholism, pregnancy and breastfeeding also resulted in non-inclusion in the study. Following the intervention, a further interview was conducted with all participants. Participants were also disqualified if, in the past 6 months, they had experienced an increase in their level of physical activity; made changes to their dietary habits, for example, if they were consulted by a qualified nutritionist; independently made dietary changes, e.g., adopting a Mediterranean diet or transitioning to a ketogenic diet; altered their treatment plan; or initiated therapy with a new medication that has been shown to impact lipid profile or has a known pleiotropic effect (such as statins, fibrates, ezetimibe, niacin, non-selective beta-blockers, metformin, sodium–glucosium-like transporter 2 (SGLT2) inhibitors, or ursodeoxycholic acid). In addition, patients were excluded if they had a history of coronary or stroke events or if they had undergone a serious infection during the intervention.

### 2.2. Evaluation of Arteriosclerotic Plaque

The carotid arteries were examined in the extracranial region using B-mode and color Doppler ultrasonography with a linear probe at a frequency of 7.5–10 MHz on a Hitachi Aloka F37 ultrasound machine (Kadoma-shi, Japan). To confirm the existence of atherosclerotic plaque in the carotid artery, we used a threshold of a C-IMT complex thickness greater than 1.5 mm or the presence of atherosclerotic plaque.

### 2.3. Anthropometry and Serum Arteriosclerotic Markers Analysis

Measurements were conducted prior to study enrollment and again after 6 months of treatment under the supervision of a physician. Standard techniques were used to assess body weight and height, and body mass index (BMI) was computed in kilograms per square meter (kg/m^2^). Measurements of waist and hip circumferences were taken at the standard anatomical positions, and the waist-to-hip ratio (WHR) was calculated. The Omron M400 Intelli IT automated device (Mannheim, Germany) was used to measure arterial blood pressure (BP) twice in the sitting position. Researchers estimated the eGFR using the CKD-EPI formula, and they reported the results in mL/min/1.73 m^2^. Standard laboratory tests were conducted in the accredited laboratory, and venous blood samples were taken after a 12 h fasting period at 8 a.m., both before and after 180 days of treatment. The chemokine and adhesion molecules serum levels were evaluated using enzyme-linked immunosorbent assay (ELISA) kits that were commercially available: MCP-1: (873.030.192, intra-assay CV%—1.8% and inter-assay CV%—3.7%), ICAM-1: (850.540.192, intra-assay CV%—1.93% and inter-assay CV%—6.04%), and L-Selectin: (850.650.192, intra-assay CV%—5.5%), all from Diaclone, (Besancon Cedex, France). To avoid the freeze–thaw effect, each experiment utilized a distinct kit and was conducted on a single sample aliquot.

### 2.4. Statistical Analysis

The data were analyzed using Statistica TIBCO Software Inc. (2017) version 13.3 software (Palo Alto, CA, USA), which was obtained through a license from the Medical University of Silesia in Katowice. We employed the Shapiro–Wilk test to evaluate the normality of the distributions. The values are reported as either means and 95% confidence intervals or medians with Q1–Q3 values. The *t*-test for independent means and the t-test for dependent means were employed to compare quantitative variables. The U Mann–Whitney test was employed to compare independent variables with a non-normal distribution. We employed the Wilcoxon test to analyze the dependent variables with a normal distribution. In addition, we employed Spearman rank correlation to evaluate the association between variables. We considered a *p*-value below 0.05 to be statistically significant.

## 3. Results

### 3.1. Study and Control Group Characteristics

The examination cohort contained 50 participants with an average age of 60.8 ± 9.5 years. Overall, 70% were obese (BMI > 30 kg/m^2^). The median glycated hemoglobin was 8.75%. All participants had recognized metabolic dysfunction-associated steatotic liver disease (MASLD) and dyslipidemia. The complete list of comorbidities and drugs used by patients is presented in Table 1 and Table 2. At the time of study, the entry median levels of total cholesterol were 166.5 mg/mL, the low-density lipoprotein-cholesterol (LDL-C) was 83 mg/dL, the high-density lipoprotein-cholesterol (HDL-C) was 49.5 mg/dL, the non-HDL was 114 mg/dL and the triglycerides (TG) were 167.9 mg/dL. Respectively, the mean levels of alanine transaminase (ALT) and aspartate transaminase (AST) were 34.4 U/L and 32.5 U/L, gamma-glutamyl transpeptidase (GGTP) was 46.3 U/L, and the mean Fibrosis-4 Index (Fib-4) was 1.5.

The control group consisted of 26 sex-matched healthy volunteers with a mean age of 33.08 ± 5.45 years, including 13 (50%) women. There were no obese individuals. All subjects were not diagnosed with any concomitant diseases. The characteristics and comparison of biochemical parameters between the study and control group are presented in Table 2 and Table 3.

### 3.2. Biochemical Effect after GLP1-RA Treatment

Following treatment in the study group, we noted a statistically significant decrease in anthropometric parameters including BMI (*p* < 0.001); on average, patients lost over 5.1 kg of weight. A decreased level in fasting glucose and an average reduction of 1.12% in HbA1C (mean: 7.56%) (*p* < 0.001) were noted. Substantial differences in decreasing blood pressure for SBP (*p* < 0.001) and DPB (*p* < 0.001) were noted. In terms of the lipid profile, alterations did not reach statistical significance. However, patients experienced a decrease in LDL fraction, TG, and non-HDL cholesterol, along with an elevation in HDL fraction. Furthermore, we obtained a statistically significant decline in the fibrosis-4 score (FIB-4) (*p* < 0.001) and De-Ritis Ratio (*p* < 0.05). The detailed results are presented in Table 3.

### 3.3. Chemokine and Adhesion Molecules Level Analysis

**Figure 1:**
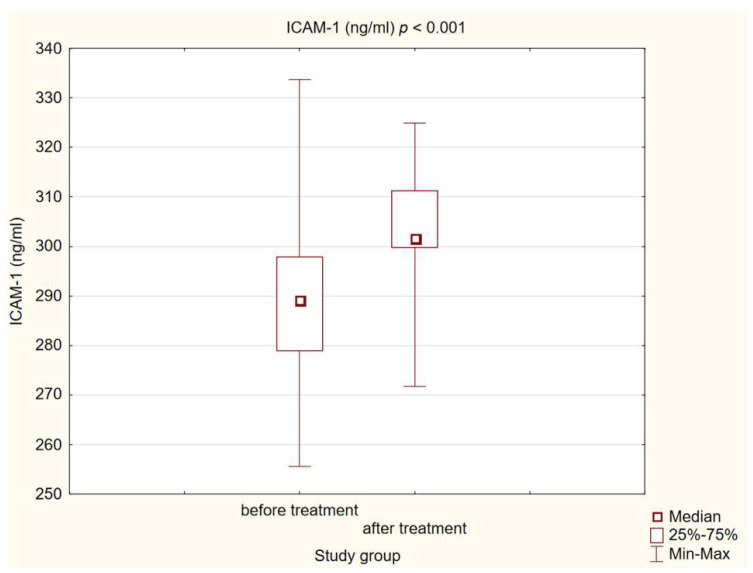
Concentration of ICAM-1 in study group before and after intervention.

**Figure 2:**
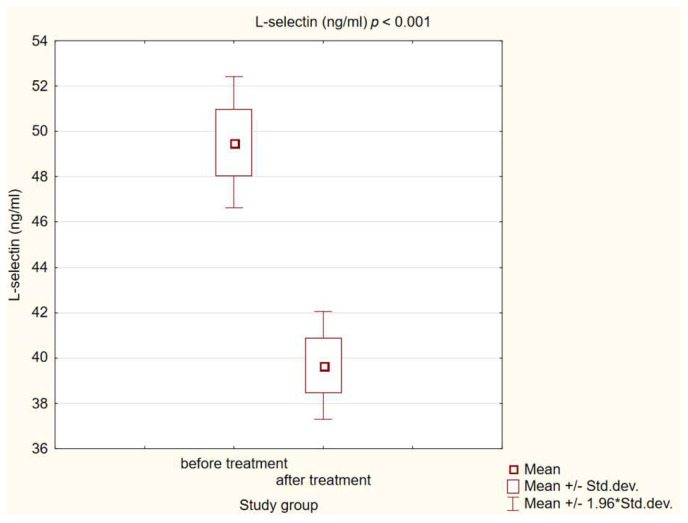
Concentration of L-selectin in study group before and after intervention.

**Figure 3:**
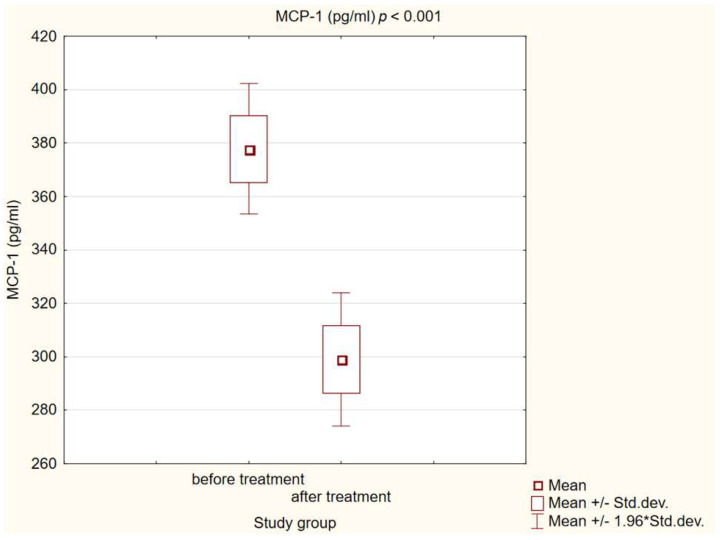
Concentration of MCP-1 in study group before and after intervention.

We observed statistically significant higher concentrations of MCP-1, ICAM-1 and L-selectin in patients with diabetes and atherosclerotic plaque before treatment compared to healthy individuals (*p* < 0.001). After a treatment intervention in our study group, we obtained a statistically significant decrease in MCP-1 and L-selectin (*p* < 0.001) concentrations, and increased ICAM-1 (*p* < 0.001). The results are presented in Figure 1, Figure 2 and Figure 3.

### 3.4. Correlation between Changes in Biochemical Parameters and Chemokine and Adhesion Molecules Levels

Additionally, we observed that changes in the concentrations of the studied adhesion molecules and chemokines do not correlate with changes in BMI, weight, lipid profile, or HbA1C value, except for ICAM-1 in terms of glycated hemoglobin. The details of the correlations we investigated are shown in Table 4 and Table 5.

## 4. Discussion

The presented study was designed to investigate the potential mechanism by which GLP-1 receptor agonists intermediate in the reduction in cardiovascular risk in patients with diabetes and atherosclerotic arterial disease. We demonstrated the beneficial effect of 180 days of therapy on biochemical and anthropometric factors associated with cardiovascular risk, such as body mass, glucose serum level or lipid profile, as well as on the concentration of chemokine and adhesion molecules responsible for recruiting monocytes to the vascular endothelium.

Numerous randomized clinical trials have demonstrated a decrease in cardiovascular risk among diabetic patients receiving GLP-1RA treatment [16,17]. This objective can be attained indirectly through the reduction in other contributing factors or directly by modulating the different molecule levels involved in atherogenesis. Our hypothesis is that GLP1-RA affects chemokine and adhesion molecules involved in monocyte recruitment to the vascular endothelium, which is one of the main pathomechanisms in the development of atherosclerosis.

Cardiovascular risk reduction using GLP1-RA could be achieved indirectly by affecting other well-documented CVD risk factors. The first of these is hyperglycemia. Diabetes type 2 is a significant risk factor for cardiovascular complications, with cardiovascular disease (CVD) being the leading cause of mortality among diabetic patients [20]. Our patients treated with GLP1-RA demonstrated benefits in terms of lower fasting glucose and glycated hemoglobin levels, comparable to those observed in randomized trials [21]. Another crucial factor to consider is dyslipidemia. Patients with diabetes are characterized by abnormalities in the lipid profile, defined as diabetic dyslipidemia, which includes high triglyceride concentrations, a decrease in the HDL cholesterol fraction and the accumulation of very low-density lipoprotein (VLDL), small dense low-density lipoprotein (sdLDL), and chylomicrons. Changes in the lipid profile accelerate atherosclerotic plaque formation [22]. Numerous scientific papers including meta-analyses of studies confirm the positive effect of GLP1-RA treatment on lipid levels in patients with type 2 diabetes. Reductions in total cholesterol, LDL fraction and triglycerides were observed [23]. In our cohort, the changes in lipid profile did not reach statistical significance. However, patients experienced a reduction in LDL, TG, and non-HDL cholesterol fractions, along with an increase in HDL fractions. This may be attributed to the fact that patients in our study group had a pre-existing diagnosis of dyslipidemia and were undergoing treatment with statins (84% individuals); at the baseline, 40% of patients had target LDL cholesterol levels and 44% target triglyceride levels, and following the therapy, there was an increase in this percentage. The absence of statistical significance in lipid profile reduction among diabetic patients was also noted in the LIRAFLAME Randomized Controlled Trial (RCT). Interestingly, in this study, a similar proportion of patients received hypolipemic treatment as in our investigation [24]. Another risk factor is excessive body weight; our patients experienced a weight loss above 5 kg during follow-up. Our findings align with previous studies, and a recently published comprehensive meta-analysis further supports the effectiveness of GLP1-RA in promoting weight reduction [25,26].

In our study, we demonstrated that patients with type 2 diabetes, dyslipidemia and confirmed atherosclerotic plaque in the carotid artery have higher levels of MCP-1, ICAM-1, and L-Selectin, compared to healthy individuals (*p* < 0.001). Numerous studies corroborate our findings, confirming that these molecules are associated with an increased cardiovascular risk [13,15,27,28].

Within the context of our research, treatment with a GLP-1 receptor agonist for 180 days led to a statistically significant reduction in MCP-1 and L-selectin levels (*p* < 0.001), accompanied by an increase in ICAM-1 levels (*p* < 0.001). Moreover, in our study, we performed a Spearman correlation at the same time, showing that the decrease in MCP-1 chemokine levels in our study was not correlated with weight loss, BMI, changes in glycemia or improvements in lipid profile.

Monocyte chemoattractant protein-1 is strongly associated with macrophages localized in the atherosclerotic plaque [29]. During atherosclerotic plaque formation, accumulated oxLDL within the endothelium enhances MCP-1 synthesis, contributing to a macrophage influx to the site of inflammation and atherosclerotic plaque progression, representing one of the key stages of atherogenesis [30]. In experimental animal models, MCP-1 lacking rabbits and mice reduced the development and progression of atherosclerosis [31]. Recent scientific literature highlights MCP-1′s involvement in atherosclerotic plaque instability; elevated levels were observed in patients with vulnerable plaque. This underscores monocyte chemoattractant protein-1 as a promising marker for evaluating the risk of cardiovascular disease stemming from atherosclerosis [32]. On the other hand, a large meta-analysis involving a substantial number of patients has indicated that, even after adjusting for conventional cardiovascular risk factors, caution should be exercised in utilizing MCP-1 levels as predictors of cardiovascular endpoints [33]. Therapeutic interventions aimed at reducing cardiovascular risk involving lifestyle modification, specifically the implementation of the dietary approaches to stop a hypertension (DASH) diet, led to a notable decrease in MCP-1 levels and enhanced the stability of atherosclerotic plaques observed on CT scanning [34]. Also, exercise reduces serum levels of monocyte chemoattractant protein-1 [35]. Drugs known for their well-documented reduction in cardiovascular risk, such as statins, demonstrated a decrease in MCP-1 levels in both cellular and animal experimental models; moreover, this effect was observed to be independent of their lipid-lowering properties [36]. Wesołowska et al., in their study, validated this observation using human peripheral blood cells. Diabetic patients treated with atorvastatin exhibited lower concentrations of MCP-1 compared to patients who did not receive statin treatment [37]. In patients with coronary atherosclerotic disease confirmed using coronary angiography, 3 months of simvastatin treatment led to a statistically significant decrease in MCP-1-induced monocyte migration [38]. Regarding the effect of glucagon-like peptide-1 receptor agonists on monocyte chemoattractant protein-1 concentrations, Bułdak et al. demonstrated that exenatide resulted in a statistically significant reduction in the concentration of this chemokine in human monocytes/macrophages treated with LPS [39]. Similar observations were described in an animal model [40]. Exenatide also reduces MCP-1 levels in patients with type 2 diabetes [41]. In a small randomized controlled trial of ninety-two diabetic patients, a significant decrease in serum MCP-1 levels was achieved after 12 weeks of liraglutide treatment [42]. Mashayekhi et al. made the same observation in their RCT involving patients with obesity and pre-diabetes [43]. In this study, like ours, the decrease in chemokine levels was independent of weight loss. In this RCT in individuals with diabetes type 2, with the same follow-up time as our study—26 weeks—and the same number of participants (n = 51), liraglutide treatment statistically significantly reduced MCP-1 levels [24]. Similarly to the findings of the aforementioned studies, our own investigation revealed that 26 weeks of GLP-1 RA treatment led to a statistically significant decrease in serum MCP-1 levels among patients with diabetes, dyslipidemia, and atherosclerotic peripheral artery disease. Evidently, large randomized trials are needed to unambiguously determine the effect of MCP-1 levels on the contribution to atherosclerotic plaque vulnerability and to confirm the decline of this chemokine as a potential cardioprotective mechanism.

Intercellular adhesion molecule-1 (ICAM-1) has been identified as one of the adhesion molecules implicated in the pathogenesis of atherosclerosis due to its role in facilitating the migration of leukocytes into atherosclerotic plaques [13,14]. In our investigation, individuals with confirmed atherosclerotic plaques exhibited significantly elevated serum levels of ICAM-1 in comparison to the healthy control group. Consistent with our findings, previous research suggests that heightened ICAM-1 levels correlate with increased cardiovascular risk [12]. Notably, Santos et al. did not observe a significant discrepancy in ICAM-1 levels across varying sizes of plaque in coronary arteries; however, elevated serum levels of ICAM-1 were associated with escalated cardiovascular mortality risk and were predictive of future fatal cardiovascular events [44]. In vitro GLP1-RA treatment led to inhibited ICAM-1 protein expression [45,46,47]. Similar results were obtained for experimental animal models [48]. The findings of experimental investigations were corroborated in a human clinical trial conducted by Luna-Marco et al., wherein a one-year treatment with a GLP1 receptor agonist in diabetic patients demonstrated a reduction in ICAM-1 concentration [49]. Our results are contradictory with those researchers. In our cohort, 180 days of GLP1-RA treatment led to increased ICAM-1 serum levels. The variations in the ultimate outcome could stem from differences in the duration of observation. Similar to our study, a randomized, placebo-controlled, double-blind, parallel-group clinical trial, PET/CT—LIRAFLAME, investigated the impact of 26 weeks of liraglutide treatment on serum markers associated with atherogenesis shown in the control group; an increase in ICAM-1 levels was also observed upon completion of the treatment [24]. The inconsistency observed in these findings across various studies underscores the necessity for additional Randomized Controlled Trials to definitively ascertain the impact of GLP-RA treatment on ICAM-1 levels.

The involvement of adhesion molecules of the selectin family such as E-selectin or P-selectin in the atherosclerotic process is well known, while that of L-selectin is still under investigation [8]. Experimental studies to date on the involvement of L-selectin in atherosclerosis have been contradictory [50,51]. A large multicenter study involving 2403 patients found no association between L-selectin and cardiovascular disease overall; however, in a subgroup analysis, a slight association with CVD risk was observed in Caucasian patients [52]. In our study, patients in the study group had significantly higher L-selectin concentrations than healthy volunteers; comparable results to ours were obtained by Nomura and colleagues [53]. Recent scientific reports show the association of elevated L-selectin values with microvascular complications in type 2 diabetes individuals [54]. In our cohort, we obtained a statistically significant reduction in serum L-selectin levels after 180 days of GLP-RA therapy; to the best of our knowledge, there are no results available evaluating the effect of this group of drugs on L-selectin adhesion molecule levels. Interestingly, another group of drugs with cardioprotective potential—statins—affect the reduction in these adhesion molecules, which was confirmed in this meta-analysis by Zinellu et al. in 2021 [55].

There are various limitations that apply to our research. Firstly, the size of our control group is limited—our study includes only 50 participants. Secondly, our study suffers from the absence of a control group treated for diabetes with therapies distinct from GLP-1 receptor agonists, or a placebo or an active comparator. Another limitation of our study is the lack of knowledge regarding the baseline lipid profile values of patients before the recognition of dyslipidemia, as well as the duration of this metabolic disease. These factors could result in varying initial cardiovascular risk levels among patients. Lastly, the study was conducted as a single-center trial, involving a population solely from one region of Poland and the end points may vary based on factors such as race or environmental conditions.

## 5. Conclusions

In conclusion, our study confirmed the already-known positive effect of GLP-1RAs on metabolic and anthropometric cardiovascular risk factors. In addition, we investigated whether these drugs affect the levels of molecules responsible for the development of atherosclerosis at the stage of the adhesion of monocytes to the endothelium. We obtained a statistically significant decrease in the concentration of MCP-1 and L-selectin. This result might be accountable for the decreased cardiovascular risk observed in diabetic individuals. On the other hand, in our study group, ICAM-1 levels increased significantly after treatment; this may be attributed to the relatively short intervention duration. Additional studies, especially randomized clinical trials with long-term observation, are needed to unquestionably assess the effect of this group of drugs on the development of atherosclerotic cardiovascular disease.

## Figures and Tables

**Table 1:** Complete treatment data for the study group. ACEI—angiotensin converting enzyme inhibitor; ARB—angiotensin receptor blocker; DPP-4—dipeptidyl peptidase-4; SGLT2—sodium–glucosium-like transporter 2; n—number of participants; %—a percentage of the survey group.

**Diabetes Treatment, n (%)**	
Metformin	49 (98%)
Sulfonylurea	22 (44%)
Insulin	14 (28%)
SGLT2 inhibitors	10 (20%)
DPP-4 inhibitors	4 (8%)
**Other treatment, n (%)**	
HMG-CoA reductase inhibitor	42 (84%)
ACEI/ARB	36 (72%)
Bblokers	21 (42%)
Indapamide	12 (24%)
Fibrates	10 (20%)
Thiazide diuretics	10 (20%)
Acetylsalicylic acid	10 (20%)
Loop diuretics	8 (16%)

**Table 2:** Baseline characteristics of patients. BMI—body mass index; SBP—systolic blood pressure; WHO—World Health Organization.

	Study Group	Control Group
Number of patients, n	50	26
Age, years	60.8	33.1
Women, n (%)	27 (54%)	13 (50%)
Men, n (%)	23 (46%)	13 (50%)
Body mass, kg	99.9	69.7
Height, cm	168.7	175.3
BMI, kg/m^2^	35	22.5
Overweight n(%)	12 (24%)	5 (19%)
Obese, n (%)	35 (70%)	0
SBP, mmHg	135	122
WHO guidelines on physical activity, n (%)	20 (40%)	14 (54%)
Smokers, n (%)		
Active	9 (18%)	1 (4%)
Past	11 (22%)	12 (46%)
Alcohol abuse, %	0	0
Co-morbidity, n (%)		
Hypertension	40 (80%)	0
Chronic kidney diseases	9 (18%)	0
Thyroid diseases	8 (16%)	0
Heart failure	4 (8%)	0

**Table 3:** Comparison of biochemical effects between the control and study groups before and after intervention. ALT—alanine transaminase; AST—aspartate transaminase; BMI—body mass index; FIB-4—fibrosis-4 score; GFR—glomerular filtration rate; GGTP—gamma-glutamyl transpeptidase; HbA1C—glycated hemoglobin; HDL—high-density lipoprotein cholesterol; LDL—low-density lipoprotein cholesterol; non-HDL—non-high-density lipoprotein cholesterol; TG—triglycerides; TC—total cholesterol; statistic significance: *—comparison between study group before and after treatment *p* < 0.001; **—comparison between study group before and after treatment *p* < 0.05; ^#^—comparison between control and study group before treatment *p* < 0.001.

	Control Group	Study Group before Treatment	Study Group after Treatment
	Mean	Mean	Mean
BMI kg/m^2^	22.46 ^#^	35.02 *^,#^	33.21 *
HbA1C %	5.5 ^#^	8.68 *^,#^	7.56 *
GFR mL/min/1.73 m^2^	96.5 ^#^	69.76 ^#^	73.1
	Median	Median	Median
Glucose mg/dL	89 ^#^	160.5 *^,#^	138.3 *
TC mg/dL	173.8	166.55	169.1
LDL mg/dL	90.5	83	80.5
HDL mg/dL	64.3 ^#^	48.95 ^#^	50.65
non-HDL mg/dL	109	113.7	107.8
TG mg/dL	86.5 ^#^	167.95 ^#^	146.5
Creatinine mg/dL	0.93 ^#^	1.06 ^#^	1.04
ALT U/I	17 ^#^	25.5 ^#^	29
AST U/I	18.5 ^#^	28 ^#^	25
De Ritis ratio (ALT/ASP)	1.11	0.98 **	0.85 **
GGTP U/I	18 ^#^	37 ^#^	35
FIB-4	0.66 ^#^	1.5 ^#^	1.3 *

**Table 4:** Correlation between changes in weight, body mass index and concentration of glycated hemoglobin and concentrations of investigated molecules. HbA1C—glycated hemoglobin; ICAM-1—intercellular adhesion molecule-1; MCP-1—monocyte chemoattractant protein-1; BMI—body mass index.

	ΔHbA1C	ΔWeight	ΔBMI
ΔICAM-1	*ρ* = −0.31, *p* < 0.05	*ρ* = −0.24, *p* > 0.05	*ρ* = 0.20, *p* > 0.05
ΔL-selectine	*ρ* = 0.13, *p* > 0.05	*ρ* = 0.13, *p* > 0.05	*ρ* = 0.15, *p* > 0.05
ΔMCP-1	*ρ* = 0.15, *p* > 0.05	*ρ* = −0.014, *p* > 0.05	*ρ* = 0.01, *p* > 0.05

**Table 5:** Correlation between changes in lipid profile and investigated molecules. TCh—total cholesterol; LDL—low-density lipoprotein; HDL—high-density lipoprotein; TG—triglycerides ICAM-1—intercellular adhesion molecule-1; MCP-1—monocyte chemoattractant protein-1.

	ΔTCh	ΔLDL	Δnon-HDL	ΔHDL	ΔTG
ΔICAM-1	*ρ* = −0.11, *p* > 0.05	*ρ* = −0.06, *p* > 0.05	*ρ* = −0.06, *p* > 0.05	*ρ* = −0.001, *p* > 0.05	*ρ* = −0.16, *p* > 0.05
ΔL-selectine	*ρ* = −0.004, *p* > 0.05	*ρ* = −0.21, *p* > 0.05	*ρ* = 0.004, *p* > 0.05	*ρ* = −0.27, *p* > 0.05	*ρ* = −0.002, *p* > 0.05
ΔMCP-1	*ρ* = 0.15, *p* > 0.05	*ρ* = 0.11, *p* > 0.05	*ρ* = 0.16, *p* > 0.05	*ρ* = −0.034, *p* > 0.05	*ρ* = 0.26, *p* > 0.05

## Data Availability

Data are contained within the article.
